# Disability Divides in India: Evidence from the 2011 Census

**DOI:** 10.1371/journal.pone.0159809

**Published:** 2016-08-04

**Authors:** Nandita Saikia, Jayanta Kumar Bora, Domantas Jasilionis, Vladimir M. Shkolnikov

**Affiliations:** 1 Centre for Study of Regional Development, Jawaharlal Nehru University, New Delhi, India; 2 Public Health Foundation of India, Gurgaon, India; 3 Max Planck Institute for Demographic Research, Rostock, Germany; 4 New Economic School, Moscow, Russia; National Cardiovascular Center Hospital, JAPAN

## Abstract

**Background:**

Understanding the socioeconomic and regional divides in disability prevalence in India has considerable relevance for designing public health policies and programs.

**Objectives:**

The aim of the present study is to quantify the prevalence of disability by gender, region (rural and urban; states and districts), and caste. We also examine the association between disability prevalence and the major socio-demographic and socioeconomic characteristics of the districts in India.

**Methods:**

Age-standardized disability prevalence (ASDP) was calculated using 2011 census data and applying the WHO World Standard Population. A regression analysis was carried out to examine the association between disability prevalence and demographic and socioeconomic characteristics across districts of India.

**Results:**

The study found that ASDP varies substantially across districts and is higher among women, rural dwellers, and members of scheduled tribes (STs) and scheduled castes (SCs). The regression model showed that the disability rate in districts rises with increasing proportions of the population who are urban dwellers, aged 65 or older, members of STs, and living in dilapidated housing; and that the disability prevalence decreases with increasing proportions of the female population who are literate, and of the general population who are working and have access to safe drinking water.

**Conclusion:**

As the burden of disability falls disproportionately across geographic regions and socioeconomic groups, public health policies in India should take this variation into account. The definition of disability used in the census should be modified to generate internationally comparable estimates of disability prevalence.

## Introduction

The WHO estimates suggest that the total global number of people with disabilities has already surpassed one billion [1]. As aging and the burden of chronic diseases increasingly affect developing countries (including India and China, the world’s most populous countries), this striking number will further increase. According to the 2011 census of India, the absolute number of people over age 60 had already reached 103.8 million, whereas the oldest-old population (over age 80) had reached 11.3 million. The same census counted around 27 million people with disabilities in India. According to a UN forecast, by 2050 there will be 323 million people over age 60 in India [2]. As aging is closely associated with increasing disability prevalence, India will face important structural and financial challenges related to the huge absolute numbers of people with disabilities requiring adequate social and health care. Expected increases in the number of people with disabilities also pose challenges for sustainable development, because disability in developing countries like India is closely related to the lack of education, extreme poverty, and social exclusion [3–5]. All of these important issues require the careful monitoring and planning of financial resources, which is impossible without more comprehensive data on disability and its determinants.

International evidence on the prevalence of disability in developing countries is scarce, and often generates contradictory figures. Disability is usually defined as a physical or a mental health condition that limits a person’s ability to perform normal life activities. However, the prevalence figures largely depend on data sources and methodological approaches (definitions). The existing rough estimations from international agencies such as the UN or the World Bank suggest that 10–12% of the global population have at least one disability [6]. However, the WHO World Health Survey and the WHO Global Burden of Disease study provide higher figures (16–19%) [6]. According to the WHO World Health Survey estimation for 2002–2004, disability prevalence in India is much higher (25%) than the global average. Although the percentage of people with disabilities is lower in India than in neighboring Bangladesh (32%), it is almost twice as high as in Pakistan and Sri Lanka. It has been acknowledged that the WHO WHS and other survey-based estimates suffer from important deficiencies related to coverage, representativeness, exclusion of most vulnerable groups, and reporting biases; and that these deficiencies may seriously distort international comparisons.

For many developing countries, the only reliable data source for disability prevalence remains population censuses. Although they provide only very broad, self-reported data, the census-based disability estimates may be based on higher levels of coverage and representativeness than surveys. The census-based figures on disability are usually lower than those based on specific survey data [6], primarily because most health surveys ask a larger number of questions and more detailed questions than the census. For example, the 2001 census estimate for India, which is based on a narrower (medical) definition of disability, indicates that the total population with any kind of disability is 11.8 million, whereas the corresponding National Sample Survey (NSS) estimate is 26.5 million [7]. Despite the large discrepancy between these two figures, certain socio-demographic patterns of disability in India emerge when we examine the data from these diverse sources. For instance, previous findings clearly indicate that the prevalence of disability in India steeply increases with age [8]. Locomotor disability has been shown to be the most prevalent type of disability in India [8]. Rates of locomotor and hearing disabilities have been found to be much higher among Indian men than Indian women, while rates of seeing disabilities have been found to be higher among women than men [8–9]. Although having a disability is often associated with severe socioeconomic disadvantages and poverty, only a small fraction of the people with disabilities in India receive government assistance [5,10–13].

The population-level evidence on regional and socioeconomic differences in disability prevalence in India is scarce. Although the Registrar General of India publishes some aggregated census-based estimates of disability prevalence at the state and the district level, these estimates are often not fully comparable due to large differences in the age compositions of states and districts. In this study, we seek to fill this gap by using district-level census information. We provide uniformly calculated values of the age-standardized prevalence of disability in India in urban and rural areas, states, districts, and castes. We examine geographical patterns in order to identify the most disadvantaged districts. This information, which is crucial for formulating policies and interventions, is complemented by an ecological analysis that reveals the relationships between district-level disability prevalence and contextual (district-level) socio-demographic and socioeconomic indicators, including indicators of population aging, female literacy, urbanization, and living conditions.

## Data and Methods

### Data description

There are two major official data sources on disability in India: the census of India and the NSS [14]. Although both sources are used to provide official statistics and nationally representative estimates of the prevalence of various disabilities, they differ considerably in terms of their coverage, the definition of disability they use, and the specific types of disability they track [15]. The 2011 census, which covers the entire population of India, provides reported information on disability as a medical condition (seven types of disability and one category for multiple disabilities). Despite having many limitations, including a tendency to underestimate the prevalence of milder impairments (especially at older ages), the census remains an important resource for studying disability patterns across regions and sub-populations in the highly diverse country of India. By contrast, most survey data on disability in India cannot be used for statistically robust and representative estimations of region-specific disability due to sample size limitations. In addition, surveys tend to differ from each other in terms of the definitions and the methods they use to estimate disability. The scope of each survey depends on its particular purpose, which is often to study the determinants of particular diseases and health conditions. If we take into account the additional efforts by the Registrar General of India to improve the census guidelines, the training of the enumerators, and the media campaigns aimed at the general public, there are good reasons to believe that the 2011 census provides better data on disability than previous censuses.

Thus, this study is based predominantly on data from the 2011 census on the disability rates, the demographic characteristics, and the socioeconomic conditions of the Indian sub-populations. Conducted by the Office of Registrar General and Census Commissioner (ORGCS), The 15^th^ Indian Census was an enormous undertaking. Some 2.7 million ORGCS officials visited all of the households in 7,935 towns and 640,867 villages in 35 states and union territories [16]. Information on disability among individuals was collected through the use of the household schedule during the population enumeration phase of the 2011 census. This information was then reported by trained enumerators who followed special instructions on how to identify various types of disabilities. The most detailed disability data in this study cover 640 districts of India.

As in the past, the 2011 census used the medical model of disability. Disability was connected to concrete medical conditions and the inability to perform concrete bodily functions, such as walking/moving, hearing, seeing, and speaking. Although the medical definitions used were quite narrow and as objective as possible, because no medical examinations were carried out the census outcomes are partly dependent on the perceptions of disability of both the enumerators and the respondents. For the 2011 census, substantial efforts were made to provide the enumerators with better training than they received in 2001, and to improve the questions. Eight categories of disability were used in the 2011 census: 1) seeing disability, 2) hearing disability, 3) speech disability, 4) movement disability, 5) mental retardation, 6) mental illness, 7) any other disability, and 8) multiple disability. The categories of mental retardation, mental illness, and any other disability were used for the first time, and the seeing and hearing categories were redefined to provide greater reliability. In the seeing category, the enumerator was asked to apply a simple test to ascertain that the respondent had blurred vision. In the speech category, respondents who could speak in single words but not in sentences were redefined as having a disability. In the movement category, eight specific types of people with disabilities were distinguished, such as those who were paralytic, those who could crawl, and those who could walk with aid [17]. Our study uses aggregated age-specific data on the disabled population across the districts, the states, and the castes of India. These data were published by the ORGCS in Table C-20, “Disabled by age group and type of disability.”

### Definitions of variables

#### Outcome variable

For all 640 districts in the 35 states and union territories of India, we defined the outcome variable as the prevalence of disability that is equal to the proportion (percentage) of people living with any kind of census-recognized disability in a given district.

#### Exposure variables

Following the literature on health and disability in India, we chose the following contextual (district-level) explanatory variables:1) the proportion of the population who are female, 2) the proportion of the population who are over age 60, 3) the female literacy rate, 4) the proportion of the population who belong to the Scheduled Castes (SCs), and 5) the proportion of the population who belong to the Scheduled Tribes (STs). The Constitution of India recognizes these castes and tribes as disadvantaged groups with special status in order to facilitate their upward social mobility [18]. These population groups also have worse health outcomes than the general population [19].

Since the census of India does not provide direct information on the incomes or expenditures of these population groups, we used a couple of alternative variables: 6) the proportion of the population who were living in an urban area, and 7) the proportion of the population who were working (i.e., those who had been working for six months or more of the reference period). We measured living conditions by 8) the proportion of households with safe drinking water (i.e., access to tap water from a treated source, a covered well, a hand pump, or a tube well), 9) the proportion of households living in dilapidated housing (i.e., a house that was decaying or breaking down and could not be restored or repaired), 10) the proportion of households with no toilet facility, 11) the proportion of households with two or more rooms, 12) the proportion of households using clean fuel for cooking, and 13) the proportion of households with access to banking services.

## Statistical Analysis

In order to ensure the comparability of estimates across states, districts, and social groups, we calculated the values of age-standardized prevalence (ASDP) using the WHO World Standard Population [20]. We carried out a descriptive analysis of the outcome and exposure variables for all 640 districts. To identify potential relationships between district-specific disability prevalence and selected socio-demographic and socioeconomic (contextual) characteristics of the districts, we conducted an ecological analysis using linear OLS regression with age-standardized disability prevalence as a dependent variable. We performed multiple linear regression in STATA S.E. 12.0. Normality tests for linear (OLS) regression models confirmed that the residuals follow a normal distribution. We used ArcGIS software to draw a district-level map of age-standardized disability prevalence.

## Results

Table 1 presents the absolute numbers of people with disabilities by age, sex, and types of residence in India; as counted by the 2011 census. The figures in parentheses show the percentage of each age group in the total population with any type of disability. The table indicates that the absolute number of people with disabilities is as high as 26.8 million, and that around 70% of all people with disabilities live in rural areas. Although the absolute number of men with disabilities is higher than the absolute number of women with disabilities, this gender gap disappears and even reverses at older ages.

**Table 1:** Absolute numbers of individuals with disability by age, sex, and urban and rural residence in India in 2011.

	Rural		Urban		Total	
Age	Male	Female	Male	Female	Male	Female
0–9	1270819 (12.2)	1055345(12.83)	501130(10.94)	419577(11.65)	1771949(11.82)	1474922 (12.47)
10–59	7033331(67.57)	5087384(61.86)	3391010(74.07)	2537966(70.48)	10424341(69.55)	7625350(64.48)
60 and above	2104018(20.21)	2081024(25.30)	685894(14.98)	643059(17.85)	2789912 (18.61)	2724083 (23.03)
Total	10408168(100.0)	8223753(100.0)	4578034(100.0)	3600602(100.0)	14986202(100.0)	11824355(100.0)

Table 2 provides the ASDP rates for Indian sub-populations, categorized by type of residence, social group, and 35 states and union territories. ASDP is marginally higher among males than among females, is higher in the rural than in the urban populations, and is higher among the SCs and the STs than among the other sub-populations. Finally, there are considerable disparities in ASDP between the states: the highest values are observed in Jammu and Kashmir in the north and in Odisha, Andhra Pradesh, Maharashtra, and Chhattisgarh in the east-center; and the lowest values are observed in the capital of Delhi, as well as in Tamil Nadu, Gujarat, and Karnataka in the south and Assam in the northeast.

**Table 2:** Age-standardized disability prevalence for Indian sub-populations and states (in %).

	Male	Female		Male	Female
**India**	2.60	2.16	Maharashtra	3.02	2.38
**Type of residence**			Odisha	3.40	2.96
Rural	2.66	2.20	Punjab	2.66	2.11
Urban	2.46	2.07	Rajasthan	3.00	2.67
**Social group**			Tamil Nadu	1.83	1.45
Scheduled Caste	2.98	2.44	Uttar Pradesh	2.46	2.03
Scheduled Tribe	2.58	2.27	West Bengal	2.53	2.12
All others	1.92	1.59	Uttarakhand	2.20	1.81
**States**			Goa	2.39	2.20
Andhra Pradesh	3.04	2.56	Arunachal Pradesh	2.56	2.54
Assam	1.88	1.77	Manipur	2.20	1.94
Bihar	2.72	2.15	Meghalaya	1.85	1.71
Chhattisgarh	3.07	2.67	Mizoram	1.75	1.55
Delhi	1.76	1.45	Nagaland	2.07	1.97
Gujarat	2.10	1.75	Sikkim	3.61	3.68
Haryana	2.58	2.11	Tripura	2.05	1.75
Himachal Pradesh	2.60	2.08	Andaman & Nicobar islands	2.20	1.85
Jammu & Kashmir	3.54	3.08	Lakshadweep	2.72	2.66
Jharkhand	2.89	2.46	Chandigarh	1.67	1.42
Karnataka	2.42	2.02	Puducherry	2.79	2.21
Kerala	2.44	1.99	Dadra & Nagar Haveli	1.28	1.16
Madhya Pradesh	2.65	2.10	Daman & Diu	1.21	1.16

Compared to the state-level data, the district-level (640 units) data provide us with a much more detailed and nuanced picture, and more opportunity for ecological analysis. In 2011, the district-level variation in ASDP was 35% higher than the state-level variation in ASDP. In terms of the max-min range, the contrast between the two levels of data aggregation was even greater. For males, ASDP ranged from 1% to 6.3% by district and from 1.2% to 3.6% by state. For females, ASDP ranged from 1% to 6% by district and from 1.2% to 3.6% by state.

Table 3 shows the descriptive statistics of the outcome and the exposure variables for 640 districts. The average value of the outcome variables (percentage of people with disabilities) is 2.2%, and the values vary between 0.8% and 4.5%. All of the socioeconomic and socio-demographic characteristics examined vary substantially across districts. For example, the proportion of the population who are members of STs ranges from a minimum of 0% to a maximum of 98.6%, while the proportion of the population who are members of SCs ranges from 0% to 50.2%. The district-level female literacy rate is about 55% on average, and varies from 24.3% to 88.6%.

**Table 3:** Descriptive statistics for outcome and explanatory variables across 640 districts of India in 2011(in %).

Variables	Minimum	Maximum	Mean	SD
***Outcome variable***				
Proportion of males with disabilities	1.02	6.24	2.58	0.66
Proportion of females with disabilities	1.03	6.03	2.17	0.63
***Demographic variables***				
Proportion of people aged 60+	2.46	17.82	8.34	2.06
Proportion of females	34.79	54.22	48.55	1.64
***Socioeconomic variables***				
Proportion of ST	0.00	98.58	17.70	26.97
Proportion of SC	0.00	50.17	14.86	9.13
Proportion of female literacy	24.25	88.62	55.24	12.41
Proportion of urban population	0.00	100.00	26.40	21.11
Proportion of main workers	30.65	96.40	73.28	12.65
Proportion of households with safe drinking water	8.60	99.60	70.68	19.95
Proportion of household in dilapidated buildings	0.20	17.70	5.03	3.12
Proportion of households with 2 or more dwelling rooms	11.90	96.50	62.72	15.45
Proportion of households with clean fuel for cooking	0.70	92.40	25.52	20.05
Proportion of households accessing banking services	10.50	93.90	58.01	16.97
Proportion of households with no toilet facility within the premises	1.10	94.40	53.63	26.30

**Fig 1:**
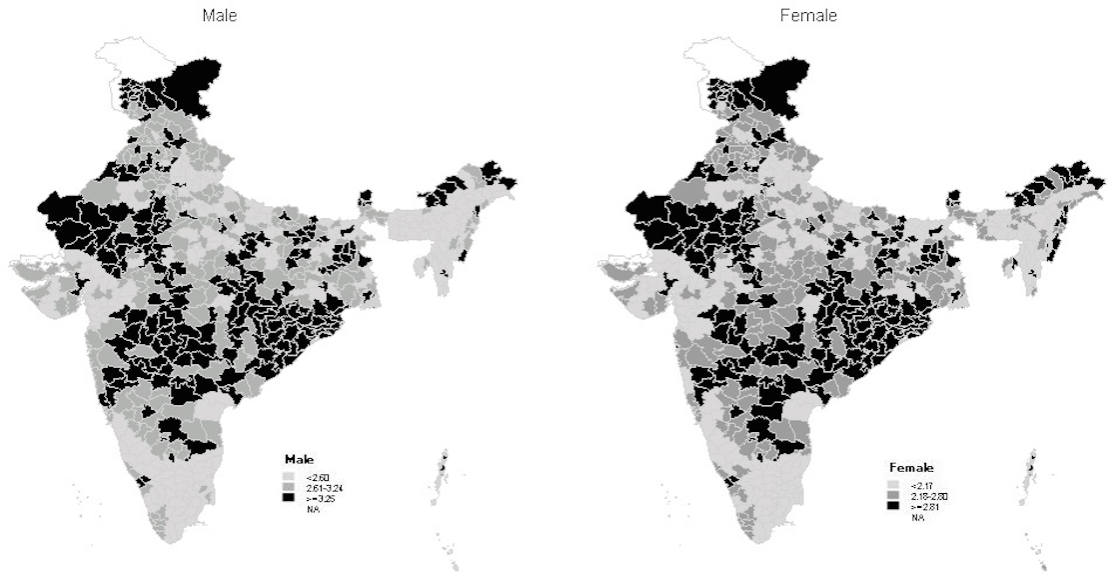
Age-standardized disability prevalence for Indian males and females in 2011.

Fig 1 and S1 File presents the spatial picture of district-level disability prevalence. The male and the female geographical patterns are very similar (the Pearson’s *r* between male and female ASDP vectors equal to 0.95). There are several continuous or nearly continuous spatial zones of higher and lower disability prevalence. A large area of high disability prevalence spreads from the districts of Odisha in the east to the southern part of Maharashtra in the west. Smaller clusters of high disability prevalence are seen in the north (Rajasthan, western part of Jammu and Kashmir, parts of Punjab and Haryana) and northeast (most of Arunachal Pradesh and Sikkim). Clusters of relatively low disability prevalence are observed in the south (Tamil Nadu, parts of Kerala, Karnataka), center-west (Gujarat and a few districts of Maharashtra), and center-north (Uttar Pradesh, most of Bihar).

Table 4 presents the outcomes of the linear regression model that assesses the relationships between the selected socio-demographic and socioeconomic contextual district-level characteristics and the age-standardized disability prevalence rates across districts. Due to the very strong correlation between the male and the female ASDP rates, the total (males and females combined) disability prevalence rate is chosen as the left-hand-side variable. The model’s outcomes suggest that the proportion of the population over age 60 and the proportion of the population who are females are the two demographic characteristics that are significantly associated with ASDP.

**Table 4:** Results of the linear regression model assessing the demographic and socioeconomic determinants of the disability prevalence variation across districts of India in 2011.

Variables	β	*p*-values	[95% Conf. Interval]
**Demographic**			
Proportion of females	-0.060	0.000	(-0.090, -0.030)
Proportion of people aged 60+	0.155	0.000	(0.122, 0.186)
**Socioeconomic**			
Proportion of ST	0.003	0.030	(0.000, 0.004)
Proportion of SC	-0.002	0.446	(-0.008, 0.003)
Proportion of female literacy	-0.013	0.000	(-0.019, -0.007)
Proportion of urban population	0.008	0.000	(0.004, 0.012)
Proportion of main workers	-0.013	0.000	(-0.017, -0.008)
Proportion of households with safe drinking water	-0.003	0.015	(-0.005, -0.000)
Proportion of households in dilapidated buildings	0.040	0.000	(0.023, 0.056)
Proportion of households with 2 or more dwelling rooms	0.001	0.673	(-0.002, 0.003)
Proportion of households with clean fuel for cooking	0.001	0.451	(-0.001, 0.004)
Proportion of households accessing banking services	0.001	0.799	(-0.004, 0.005)
Proportion of households with no toilet facility within the premises	0.001	0.467	(-0.001, 0.004)
**R**^2^	**0.2024**		
**Adjusted R**^2^	**0.1898**		

Although the districts where a relatively large proportion of the population are members of STs also tend to have higher disability rates, this is not the case for districts where a large proportion of the population are members of SCs. Districts with high female literacy rates and a relatively large proportion of the population who work have lower disability prevalence. At the same time, districts that are more urbanized tend to have higher disability rates.

Among the variables that reflect average household living conditions, we found the proportion of households with safe drinking water and the proportion of households in dilapidated housing to be statistically significantly associated with disability prevalence: an increase in the proportion of households with safe drinking water is linked to a decrease in disability prevalence, while an increase in the proportion of households in dilapidated houses is linked to an increase in the disability variable. We found no statistically significant associations between disability prevalence and the proportion of households without a toilet, the proportion of households with two or more dwelling rooms, the proportion of households using clean fuel for cooking, or the proportion of households with access to banking services.

A 1% additive change in the proportion of the population who are over age 60, the proportion of the population who are females, and the proportion of households in dilapidated houses produce the first-, second-, and third-largest changes in the dependent ASDP variable.

## Discussion

In this study, we examined the prevalence of disability in India. We computed the values of the age-standardized prevalence of disability among men and women, urban and rural dwellers, members of social-ethnic groups (STs, SCs, others), the populations of 35 states, and the populations of all 640 districts within these states. We mapped spatial patterns of disability and revealed meaningful associations across space between disability and certain demographic characteristics and socioeconomic conditions. To the best of our knowledge, there has up to now been no systematic study of the disparities in the prevalence of disability in India that examined in detail the demographic, socioeconomic, and geographic characteristics of the population with disabilities. We thus believe that this study adds important information to the existing literature.

Our study has three limitations. First, the definition of disability used in this study is based on the narrowly oriented medical model, and cannot be compared with the findings of other studies that used the International Classification of Functioning, Disability and Health (ICF) definition. This is because the ICF model of the WHO combines disability due to impairment, activity limitations, and participation restrictions [7,21]. Second, the census information on disability has a substantial subjective component, as it depends on the perception of disability of both the respondent and the interviewer. This implies that the rates of disability reported may not be fully comparable across regions with different religious and ethno-cultural features. This inherent subjectivity, combined with our failure to find any statistically significant correlation between state-level disability and mortality (analysis not shown in the paper), calls to mind Amartya Sen’s observation that there could be biases in the morbidity data in India that are caused by variation in people’s perceptions of illness depending on the local availability of medical services and culturally perceived norms [22]. Finally, we assessed the relationship between the disability and the explanatory variables at the aggregate (district) level. Hence, the inferences drawn from the regression model hold only for districts and not for individuals. In addition, the cross-sectional nature of the data suggest that the relationships identified by the statistical model cannot be seen as causal. Nevertheless, the analyses provide insights into the disability burden and the differentials in India based on the recent census data, and thus provide an exhaustive (or nearly exhaustive) enumeration of the people with disabilities.

As the study was based on the conservative “medical” definition, milder forms of disability and disabilities caused by local environmental barriers may be underestimated. Nevertheless, we found large geographical and social disparities in the prevalence of disability across India; i.e., that the burden of disability is disproportionately concentrated in certain disadvantaged regions and districts, and in certain population groups. The highest levels of age-standardized disability prevalence were found in the far north, the northeast, the center, and the west-center of the country. Lower levels of disability were seen in Delhi, in the south and the west of the country, and (surprisingly) in Assam in the north-east and in Uttar Pradesh in the north-center of the country. Within India, Assam and Uttar Pradesh also have unusually high levels of mortality[23].

In line with previous studies in developed and developing countries, we found associations between district-level disability and the corresponding demographic and socioeconomic contextual characteristics. Not surprisingly, ASDP tends to be higher in districts where the proportion of older people is relatively high. The modeling results also show that socioeconomic disadvantages at the district level, such as poor household living conditions and a high proportion of the population who are members of deprived STs generally contribute to a higher disability prevalence. The notable exception is our finding that the proportion the population who are members of SCs has no relationship to district-level disability levels. The latter result may arise from a kind of ecological fallacy.

One important finding of the present study is that men appear to have higher levels of disability in terms of both absolute numbers and age-standardized prevalence. This result contradicts evidence from other countries that women have higher disability levels despite also having higher survival rates [1,24–26]. There are several possible explanations for the unexpected pattern observed in India. First, most of the previous studies that reported higher levels of disability among women were mainly based on reports of the ability to perform activities of daily living (ADLs) or functional assessments. These studies found that women tend to have chronic conditions that are less severe and less lethal than of the conditions men tend to have [27–28]. Thus, the sex pattern observed in this study might be attributable to the underreporting of milder forms of disability in the census. Second, the male disadvantage in disability prevalence is most pronounced at younger ages and reverses at older ages, when disability prevalence is especially high [29–31]. Third, even if we assume that the census officials did their best to count every individual with any disability condition, we cannot totally reject the hypothesis that the number of women with disabilities may have been underreported because of the stigma of disability and the tendency to overlook women. Although we have not found any study that addresses the under-enumeration of females in the 2011 census, a few studies have shown that the 1991 census under-enumerated females, especially marginalized females (widows, elderly women, etc.) [32]. Finally, because of gender discrimination in nutrition and health care, there is excess mortality among females under age five in India [16]. It is possible that female children with disabilities experience a double burden of discrimination, and are thus subject to a higher risk of death than male children or girls without disabilities. As expected, we found that in India, as in other developing countries, a higher female literacy rate is associated with a somewhat lower disability prevalence rate [19].

## Conclusion

The findings of this study are relevant for the design of public health policies and programs in India. There are large disparities in the prevalence of disability across geographic areas and socioeconomic strata. Therefore, public health policies that target specific regions or groups may be needed. In particular, the government should take special measures to address the higher burden of disability in the majority of districts in Arunachal Pradesh, Chhattisgarh, Jammu and Kashmir, Odisha, Maharashtra, Rajasthan, and Sikkim. It is also crucial that the government steps up its efforts to improve the socioeconomic conditions of the underprivileged segments of the population. As older people are especially likely to develop disabilities, and the aging of the population is already underway in several states, public health policy-makers should seek to address these growing disability care needs, while taking into account the direct and indirect effects of the presence in families of people with disabilities on the other family members.

Future research should also examine the reporting and the possible understatement of disability across India, and in certain regions in particular. The results of this study suggest that the definition of disability used in the census of India should be modified to reflect the broader definition of the WHO in order to produce internationally comparable results.

Additional micro-level research on the current status of disability care should be carried out in regions and population groups with a higher burden of age-standardized disability. Special attention should be paid to health care utilization by individuals with or without disabilities, especially those in socially disadvantaged groups.

## Supporting Information

S1 File (XLSX)
