# Correction: Disability Divides in India: Evidence from the 2011 Census

**DOI:** 10.1371/journal.pone.0172596

**Published:** 2017-02-15

**Authors:** Nandita Saikia, Jayanta Kumar Bora, Domantas Jasilionis, Vladimir M. Shkolnikov

The resolution of Fig 1 is too low to view the map legends clearly. Please view the corrected Fig 1 here.

**Fig 1:**
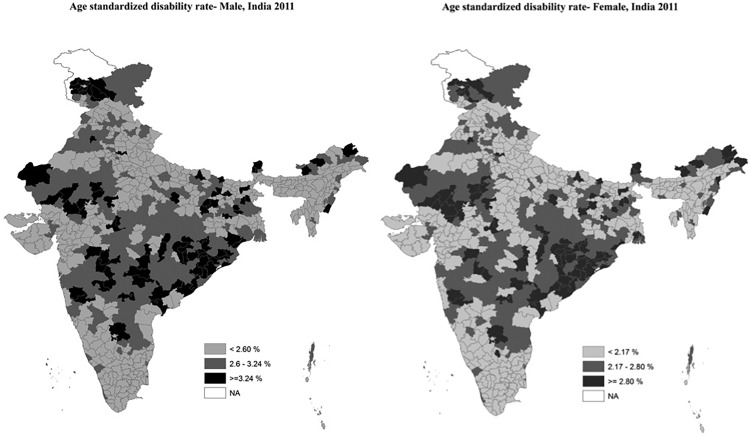
Age-standardized disability prevalence for Indian males and females in 2011.
